# 1,1-Difluoroethane Detection Time in Blood after Inhalation Abuse Estimated by Monte Carlo PBPK Modeling

**DOI:** 10.3390/pharmaceutics12100997

**Published:** 2020-10-20

**Authors:** Raul Huet, Gunnar Johanson

**Affiliations:** 1 Department of Psychiatry, Volunteer Faculty, University of Missouri-Kansas City School of Medicine, 2411 Holmes Street, Kansas City, MO 64110, USA; rahuet@sbcglobal.net; 2 Department of Psychiatry, Volunteer Faculty, University of Kansas School of Medicine, 3901 Rainbow Blvd., Kansas City, KS 66160, USA; 3 Toxicology and Risk Assessment, Integrative Toxicology, Institute of Environmental Medicine, Karolinska Institutet, Box 210, SE-17177 Stockholm, Sweden

**Keywords:** aerosol spray, air duster, compressed gas, detection limit in blood, hydrofluorocarbon, maximum detection time in blood, Monte Carlo simulation, physiologically based pharmacokinetic model, propellant, toxicokinetics

## Abstract

(1) Background: Inhalant abuse and misuse are still widespread problems. 1,1-Difluoroethane abuse is reported to be potentially fatal and to cause acute and chronic adverse health effects. Lab testing for difluoroethane is seldom done, partly because the maximum detection time (MDT) is unknown. We sought to reliably estimate the MDT of difluoroethane in blood after inhalation abuse; (2) Methods: MDT were estimated for the adult male American population using a physiologically based pharmacokinetic (PBPK) model and abuse patterns detailed by two individuals. Based on sensitivity analyses, variability in huffing pattern and body mass index was introduced in the model by Monte Carlo simulation; (3) Results: With a detection limit of 0.14 mg/L, the median MDT was estimated to be 10.5 h (5th–95th percentile 7.8–12.8 h) after the 2-h abuse scenario and 13.5 h (10.5–15.8 h) after the 6-h scenario. The ranges reflect variability in body mass index and hence amount of body fat; (4) Conclusions: Our simulations suggest that the MDT of difluoroethane in blood after abuse ranges from 7.8 to 15.8 h. Although shorter compared to many other drugs, these MDT are sufficient to allow for testing several hours after suspected intoxication in a patient.

## 1. Introduction

Inhalant abuse continues to be a problem, especially among adolescents. In 2019, the percentage among 8th, 10th, and 12th graders in the USA that reported use of an inhalant in the past year was 4.7%, 2.8%, and 1.9%, respectively [1]. In 2019, the percentage among age groups 12–17, 18–26, and 26 years or older in the USA that reported use of an inhalant in the past year was 3.0%, 1.7%, and 0.4%, respectively [2]. This paper focuses on one commonly used spray can propellant, namely 1,1-difluoroethane (DFE, HFC 152a). The interest emerges as in the clinical practice of addiction psychiatry one sometimes encounters patients who use computer keyboard air duster cleaner containing essentially 100% DFE. The percent of inhalant users who use computer keyboard air duster cleaner containing 1,1-difluoroethane (DFE, HFC 152a) is not reported in the medical literature. However, in 2000 and 2001, among American 12–17-year-olds who reported any lifetime inhalant use, 18.8% had used aerosol sprays [3]. Importantly, inhalant use is to be distinguished from Diagnostic and Statistical Manual-4 or 5 (DSM-4 or DSM-5) defined inhalant use disorder. In 2000 and 2001, only 10% of 12–17-year-old USA teens who reported past-year use were found to satisfy past year DSM-4 criteria for inhalant abuse or dependence [3]. Likewise, in 2001 and 2002, only 19% of USA adults who reported lifetime inhalant use met lifetime DSM-4 criteria for inhalant abuse or dependence [4]. Thus, inhalant misuse is far more common than indicated by the prevalence of inhalant use disorder.

Unfortunately, there is only limited laboratory testing performed to document recent abuse of inhalants. In drug addiction treatment, a general problem with many of the inhalants, in particular those used as propellants, is that most have a high vapor pressure and low blood solubility and are therefore rapidly cleared from the body with initial half times in the blood of a few minutes. Therefore, abuse is not easily detected as sampling time compared to exposure time dictates the likelihood of detecting recent use. Assuming that a maximum detection time (MDT) of reasonable duration is known, inpatient and outpatient inhalant use disorder treatment programs would enormously benefit for two reasons: (1) it would help the provider detect relapse or continued abuse, and (2) it would likely increase the motivation of patients to remain abstinent.

There are various methods for categorizing inhalants and one such method used by the National Institute of Drug Abuse in the USA lists these categories as aerosol sprays, solvents, gases, and nitrites [5]. Some of the hazardous chemicals in these inhalants are amyl nitrite, butyl nitrite, benzene, butane, propane, hydrochlorofluorocarbons (HCFCs), chlorofluorocarbons (CFCs), hydrofluorocarbons (HFCs), methylene chloride, nitrous oxide, toluene, acetone, methylethylketone, hexane, pentane, xylene, diethyl ether, halothane, chloroform, and enflurane [6]. Some of the street terms used in the USA to describe the method of inhalant use include sniffing, snorting, dusting, glading, bagging, huffing, and spraying [7,8]. Some simply inhale from balloons filled with nitrous oxide.

DFE is a colorless, flammable gas at room temperature and normal atmospheric pressure. It has a slight ethereal odor and a historically reported low toxicity and is one of the major HFCs that have replaced HCFCs and CFCs in, e.g., refrigerators and foam blowing, due to a lower impact on the ozone layer. DFE is also commonly found in electronic cleaning products, e.g., computer keyboard air duster cleaner, and other consumer aerosol products [9,10].

Rats exposed to 3000 ppm DFE for 4 h showed no signs of adverse effect [11]. The substance may, however, induce cardiac sensitization at higher exposure levels (150,000 ppm). No evidence of toxicity or carcinogenicity was found in a 2-year rat inhalation study with exposures up to 25,000 ppm [9]. No maternal or developmental toxicity was noted in rats at 50,000 ppm, the highest level tested [12]. The relatively lower toxicity of DFE contrasts that of 1,2-diflouroethane which is highly toxic to rats. The toxicity is thought to be mediated by fluoroacetate, a metabolite of 1,2-difluoroethane but not of DFE [11]. No animal toxicokinetic data on DFE were found in the scientific literature, except one study with rats, where the time course was followed in blood, brain, heart, liver, and kidney up to 15 min after exposure at levels corresponding to abuse [13].

DFE has become a substance of abuse due to its ease of access and because it causes euphoria when inhaled [14]. Fatal cardiac arrhythmias after DFE inhalation have been reported [15] and have been associated with the “sudden sniffing death syndrome” which can occur even the first time it is inhaled [16,17,18]. It has been reported to be the leading cause of fatalities related to inhalant use [8]. DFE and other inhalants reportedly “sensitize” the myocardium to epinephrine and when this catecholamine is produced by a sudden fright or other stimulus, it can lead to a fatal arrhythmia. Death can also result from motor vehicle accidents, falls, or drowning due to confusion, drowsiness or loss of consciousness while intoxicated with DFE [19]. Airway compromise due to frostbite caused by inhaled DFE can potentially lead to death as can oxygen displacement [20,21]. Attempted suicide from DFE inhalation has been reported [22,23]. The Florida State Medical Examiners Commission found that there were 49 statewide deaths caused by inhalants in 2018 of which 83% involved halogenated inhalants, especially DFE [24]. Reported acute health effects of DFE inhalation in nonfatal cases include auditory and visual hallucinations and delusions [25], mania with psychosis [26], disorientation, poor judgement and disinhibition, tremors, ataxia [23], generalized tonic clonic seizure activity [27], dyspnea on exertion [23], pulmonary irritation and cough [28], frostbite burns of skin [20,29], cutaneous burn injury by hydrofluoric acid from thermal degradation of DFE [30], cutaneous flame burns from fire ignition of DFE [30], angioedema [31], palpitations and irregular pulse, chest pain, pneumopericardium [29], cardiomyopathy [32,33], toxic myocarditis [23], nausea and vomiting, acute liver injury and fulminant hepatitis [23,33,34], rhabdomyolysis and acute renal failure [22,33,34]. Some reported long-term health effects include skeletal fluorosis [35] and possibly chronic kidney disease [22]. Though not specific to DFE abuse, inhalant abusers in general have higher rates of major depression, suicidal ideation and attempts, and other substance use disorders than nonusers of inhalants [36,37].

A decade ago, we conducted a controlled human exposure study to determine the toxicokinetics of inhaled DFE [10]. Healthy volunteers were exposed for 2 h to 0, 200, or 1000 ppm DFE during light exercise. As expected, symptom ratings and changes in inflammatory markers in blood revealed no exposure related effects. Capillary blood, urine, and exhaled air were sampled up to 22 h post exposure and analyzed for DFE, while fluoride and other potential metabolites were analyzed in urine. DFE showed a very low respiratory uptake, a fast increase in blood levels within the first few minutes and a rapid post-exposure decrease. The DFE concentrations in blood and exhaled air were proportional to the exposure concentrations, suggesting linear, first-order kinetics at least up to 1000 ppm. About 20 µmol excess (compared to control) fluoride was excreted in urine after exposure to 1000 ppm. This corresponds to 0.013% of inhaled DFE on a molar basis. No fluorine-containing metabolites were detected in urine. Overall, the results suggest little or negligible metabolism of DFE in humans. The time courses of DFE in blood and exhaled air were well described with a physiologically based pharmacokinetic (PBPK) model [10]. PBPK models are used to calculate the uptake and disposition of a chemical in the body from relevant quantitative data on physiology and anatomy (e.g., organ volumes and composition, blood flows, and lung ventilation) chemical-dependent factors (e.g., blood:air and tissue:blood partition coefficients), and metabolic rates of the chemical. This allows for prediction of the disposition for new circumstances, such as different exposure scenarios, body build (obese vs. slim), or physical workload [38].

DFE can be tested for in blood after inhalation abuse but an MDT after abuse has never been reported in the medical or toxicology literature, albeit, it has been detected in blood tests up to almost 3 h after abuse in nonfatal motor vehicle accidents [39]. The present study aimed to more closely estimate the MDT of DFE in blood after inhalation abuse, by using the previously developed PBPK model [10], combined with patients’ data on abuse behavior, information on the analytical detection limit, and with Monte Carlo simulation to account for variability in inhalation patterns and body composition. The PBPK-Monte Carlo approach is an attractive alternative to empirical studies among patients as well as experimental exposures of volunteers which would require more resources and be ethically questionable.

## 2. Materials and Methods

### 2.1. PBPK Model

**Figure 1:**
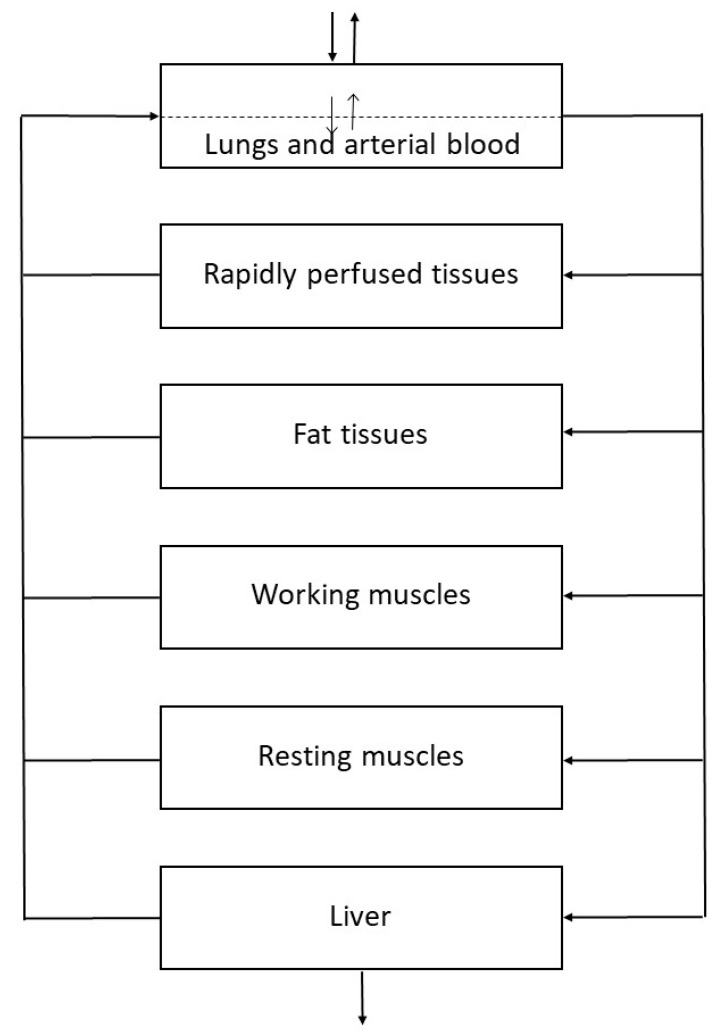
Physiologically based pharmacokinetic model used to simulate the inhalation toxicokinetics of 1,1-difluoroethane in humans. Reproduced with permission from [41]; Published by Elsevier, 2014.

In the aforementioned study [10], we applied a PBPK model to further explore the experimental toxicokinetic data. The model described the data well and was consistent with low or even zero metabolism, where the low uptake during exposure is explained by storage of DFE in adipose tissues and the slower second phase after exposure is due to washout from adipose tissue. The model structure is shown in Figure 1. A special feature of the model is that it distinguished resting muscle from working muscle in two separate compartments. This PBPK design was developed by Johanson and Näslund [40] for experimental inhalation studies performed with exercise on a bicycle ergometer, with leg muscles working and muscles in the upper part of the body at rest. In the present study, muscles were treated as a single compartment. The PBPK model parameters (Table 1) were in line with those we previously applied for HFCs including DFE [10,41].

In the Monte Carlo simulations, the volumes and flow parameters in the PBPK model were scaled from physical workload, body weight, and height according to equations given by Nihlén and Johanson [42]. Near resting condition was assumed during and after the abuse session, hence the workload was set to 10 W.

**Figure 2:**
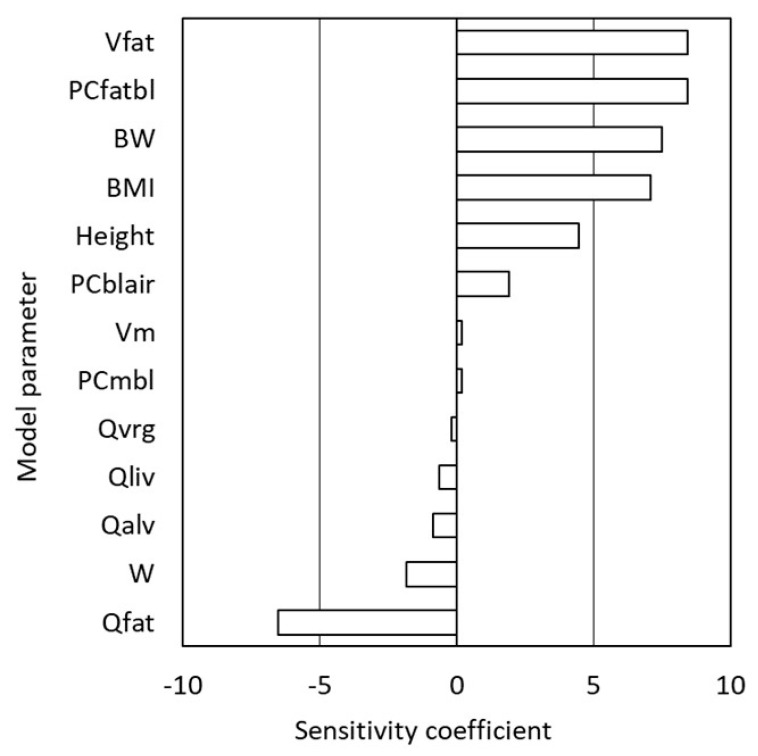
Local sensitivity analysis of the PBPK model for 1,1-difluoroethane (DFE). Normalized sensitivity coefficients (ratios between percentage change in DFE and percentage change in model parameter) were calculated for DFE in mixed venous blood at 24 h with a standard man (*BMI* 24.2) and scenario Y. Parameters with sensitivity coefficients between −0.1 and 0.1 are not shown. *Vf*—volume of fat compartment; *PCfb*—fat:blood partition coefficient; *BW*—body weight; *BMI*—body mass index; *PCba*—blood:air partition coefficient; *Vm*—volume of muscle compartment; *PCmb*—muscle:blood partition coefficient; *Qvrg*—blood flow of rapidly perfused organ; *Qliv*—liver blood flow; *Qalv*—alveolar ventilation; *W*—physical workload; *Qfat*—fat blood flow.

A local sensitivity was performed (Figure 2), showing that the most influential factors were indirectly (body weight, height, body mass index) or directly (fat:blood partition coefficient, volume and blood flow of the fat compartment) related to body fat. Our model uses body weight (*BW*) and height (*H*) for scaling of volumes and flows, while we only had access to demographic data on body mass index (*BMI*). The *BMI* is defined as:

*BMI* = *BW* (kg)/*H* (m) (1)

In order to allow body fat to vary and still use the scaling equations, we therefore kept *H* constant at 1.7 and calculated the *BW*, as:

*BW* = *BMI* × 1.7 (2)

The distribution in *BMI* was obtained by fitting a normal distribution to NHANES III demographic data on American men (1527 men 17 years and older) reported by Ritchey et al. [43]. These data (presented as number of men in each of four *BMI* categories) yielded a mean of 25.8 and a standard deviation of 3.43. The *BMI* distribution was truncated at 16.5 and 40 to avoid unrealistic values.

### 2.2. Exposure Scenarios

Exposure scenarios (Table 2) were derived from interviews with two individuals regarding typical keyboard air duster cleaner abuse sessions. The keyboard cleaner product used was purchased in canisters each containing 10 ounces (283.5 g) of essentially 100% DFE. Person X reported inhaling directly from the canister nozzle into the mouth. Eight canisters of keyboard cleaner were usually abused over a period of about 6 h. The typical inhalation would last for about 1.5–3 s. The inhaled DFE would then be held in the lungs for about 1–5 s. The rate of inhalations was about one every 3–5 min. Person Y reported inhaling directly from the canister nozzle into the mouth. About six or seven canisters were usually abused over a period of about 2 h. The typical inhalation would last about 5 s. The inhaled DFE would then be held in the lungs for about 10 s. The rate of inhalations was about one every 2–5 min.

### 2.3. Detection Limit(s) for DFE in Blood

According to the literature, most investigators have used head-space gas chromatography with flame ionization (GC/FID) or mass spectrometry (GC/MS). The limits of detection (LOD) and quantification (LOQ) vary widely between studies (Table 3). For example, NMS Labs tests for DFE in blood by GC/MS and states that their reporting limit (LOQ) is 0.14 mg/L [44]. Notably, levels greater than 5.4 mg/L are judged by one published report to be evidence of intentional inhalation [28].

### 2.4. Influence of BMI

The sensitivity analysis suggested a huge impact of body fat. Single simulations were first carried out for three hypothetical individuals with the same height but different *BMI*, representing a slim, normal, and obese male, using one of the exposure scenarios.

### 2.5. Monte Carlo Simulations

The PBPK model was used to simulate time courses of DFE in blood for different inhalation exposure scenarios including interindividual variability in breathing pattern and *BMI*. For each exposure scenario a range of curves (blood concentration vs. time) were obtained by Monte Carlo simulation (1000 iterations), using random sampling from uniform distributions of the inhalation parameters (duration and interval, ranges given in Table 2) and a normal distribution of *BMI* (see section PBPK model). The simulations were done with Berkeley Madonna software (v. 9.1.19) using the fourth order Runge–Kutta method. MDTs were calculated as the time from cessation of the abuse session to reach the detection limits for DFE in blood given in Table 3.

### 2.6. Ethical considerations

All experiments herein were computer-based simulations, using aggregate literature data on *BMI* and anatomical and physiological data. The only exception was the information on inhalation behavior during the abuse of DFE, used as the basis for the exposure scenarios (Section 2.2 and Table 2). These data were collected as supplementary information during clinical interviews with two patients while in treatment. Written informed consent to use the inhalation behavior data was obtained from the patients.

## 3. Results

**Figure 3:**
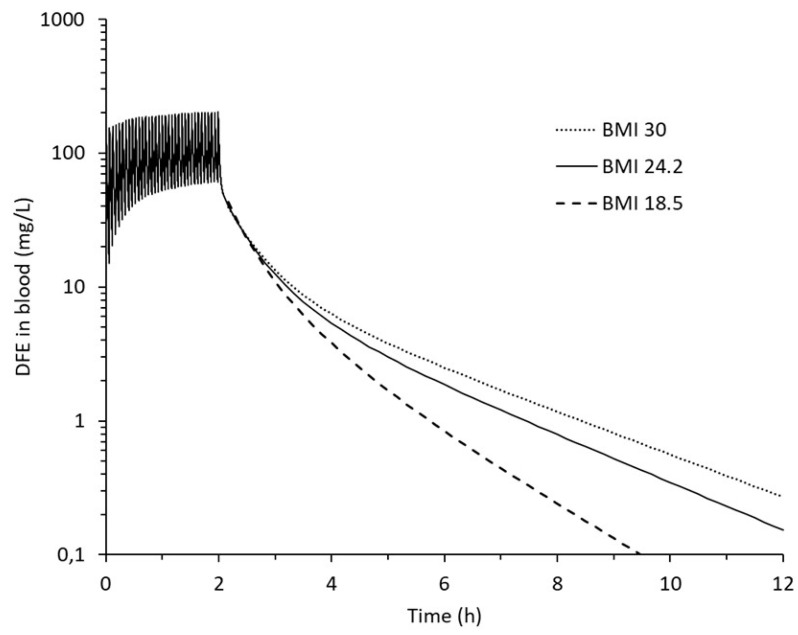
Simulated concentration of DFE in mixed venous blood during and after a 2-h abuse session illustrating the influence of body composition. The three curves represent underweight (*BMI* 18.5), normal weight (*BMI* 24.2), and overweight (*BMI* 30) individuals. The fluctuations during the abuse phase reflect repeat 10-s inhalations of 100% DFE every 3.5 min (the central estimates for scenario Y). As the fluctuations are very similar for the three, they are only shown for *BMI* 24.2.

Initial simulations (Figure 3) confirmed that *BMI*, reflecting body composition and body fat, is important for the decline in DFE in blood after the abuse session and thus affects the MDT.

These simulations further illustrate that DFE in blood typically ranges approximately 5-fold during the huffing sessions, with a range between 40 and 200 mg/L and an average of 100 mg/L for scenario Y. Three phases can be distinguished post-exposure. A fast phase is seen during the first few minutes due to washout from the highly perfused organs, i.e., the lung, VRG and liver compartments (the half time in the latter two is 0.8 min) and partial refill by DFE deposited in muscles and fat. An intermediate phase occurs for a few hours and is explained by washout from muscles (half time 41 min) and refill from fat. Lastly, a slow phase is seen, reflecting washout from fat tissue (half time 3.0 h).

**Figure 4:**
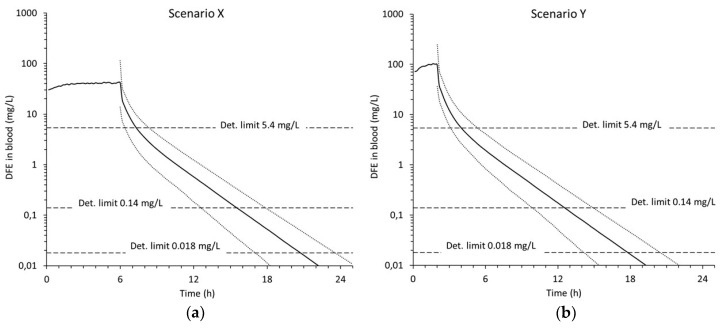
Simulated concentration of DFE in mixed venous blood for inhalation exposure scenario X (**a**) and Y (**b**). See Table 2 for scenario descriptions. Note the log scale of the concentration axes. The solid curves represent the median and the dotted ones the 5th and 95th percentiles from 1000 Monte Carlo simulations. The horizontal lines denote the three different detection limits. Fluctuations during abuse phase are not shown.

Monte Carlo simulations with variable inhalation patterns and *BMI* values are presented in Figure 4. The median concentration of DFE (smoothed to eliminate the fluctuations between huffs) reaches about 40 mg/L for scenario X and about 100 mg/L for scenario Y. The dotted curves in Figure 4 show the 5th and 95th percentiles and give an indication of the intraindividual variability. For both scenarios, the 5th–95th percentile range is about 3-fold early after the abuse session, a reflection of the interindividual variability in inhalation pattern. As time passes, this range increases to approximately 10-fold, the increase is a reflection of the interindividual variability in *BMI*/body fat.

The MDTs (medians and 5th–95th percentile ranges) obtained in the Monte Carlo simulations are given in Table 4. The MDT depends on the amount of DFE inhaled (body burden) as well as the detection limit. Thus, the median of the MDT ranges widely, from 2.0 h for the shorter exposure scenario (Y) with the highest detection limit, to 18.7 h for the longer exposure scenario (X) with the lowest detection limit. Most emphasis should be on the mid detection limit of 0.14 mg/L as this has been reported as the limit of quantitation by an accredited lab [44].

The differences in elimination rate also affect the MDT. For the highest detection limit (5.4 mg/L), the range of MDTs is about 2 h (Table 4). Here, most of the variability is due to variations in inhalation pattern. For the two lower detection limits, the range increases to 5–6 h.

## 4. Discussion

Our study focused on an abused inhalant, DFE, that is still of concern within the addiction treatment community. The main finding of our study is that DFE can be detected in blood for hours after abuse scenarios as specified in detail by two individuals in our study. With a detection limit (LOQ) of 0.14 mg/L, the predicted median MDT is 13.5 h after the 6 h abuse scenario and 10.5 h after the 2 h abuse scenario. To our knowledge, this represents the first attempt to determine the MDT of DFE in blood after inhalation abuse.

The strength of our predictions is that the toxicokinetics are fairly uncomplicated; for example, the influence of protein binding and metabolism is negligible. Thus, the used PBPK model is straightforward and can very well describe the uptake and disposition of DFE and several other HFCs as previously shown [41].

The weaknesses of our study include that the exposure scenarios are only based on interviews and only obtained from two individuals. Furthermore, there are no experimental toxicokinetic data at these high exposure levels and the toxicokinetics may well change at intoxication levels, e.g., due to changes in breathing and circulation. Inter- and intraindividual variability in lung ventilation, fat perfusion and body fat (reflected by *BMI*) may all affect the detection time. Our sensitivity analyses (Figure 2) suggest that the lung ventilation (represented by *Qalv* in Figure 2) is less influential among these factors whereas fat volume and blood flow are more important. A wide intra- and interindividual variability in fat blood flow has been demonstrated experimentally in subcutaneous abdominal adipose tissue [49] and by Bayesian estimation using a PBPK model [50]. Much of the interindividual variability in fat blood flow is explained by body build (i.e., fat volume), while short-term intraindividual variability (over a few hours) has little influence on the MDT, which is more dependent on the time-weighted average fat blood flow of each individual. Meanwhile, body fat is highly variable between individuals (but shows little intraindividual variability, at least over a few hours). We therefore believe that body fat/*BMI* is the most important among these factors, though the inclusion of fat blood flow variability in the Monte Carlo simulations would probably have resulted in slightly wider ranges of MDTs. On the other hand, MDT did not correlate with *BMI* in the Monte Carlo simulations (data not shown), suggesting that the increase in MDT with increased *BMI* (inferred by Figure 3) is masked by the much greater influence of variable inhalation patterns.

We did not find any human data on exposure levels causing central nervous system effects. In one study [13], rats were exposed to DFE for 30 s with an average calculated concentration during this time of 77,200 ppm (our calculation, based on the methodological description by the study’s authors). The rats first became significantly intoxicated at 20 s and thereafter remained sedated up to 4 min. Blood levels initially rapidly rose to 350 mg/L and declined to about 20 mg/L at 4 min. This corresponds well with the predicted range of 40–100 mg/L during abuse in our two scenarios.

Although the MDT of DFE is of relatively short duration compared to many other drugs, it is still longer than many may have imagined. Furthermore, as indicated in Table 3, the MDTs would be further prolonged by using more sensitive analytical methods of detection, for example, thermal desorption gas chromatography combined with electron capture detection. In this context, it is worth noting that blood must be handled with care all the way from sampling to analysis to minimize evaporation losses.

In conclusion, using PBPK modeling and Monte Carlo simulation, we estimate that the MDT of DFE in blood after abuse is on the order of hours, sufficient to allow testing for it even up to 8–16 h after suspected intoxication. It is welcome news in the treatment of this health problem. In turn, the hope is that this may make it possible to help the DFE abuser “make peace with their demons,” one of the principles of acceptance and commitment therapy used in many treatment programs.

## Figures and Tables

**Table 1:** Model parameter used in the physiologically based pharmacokinetic (PBPK) simulations. The presented volumes and flows correspond to those of a resting “standard man” (body weight 70 kg, height 170 cm, body mass index (*BMI*) 24.2) at near rest (physical workload 10 W).

Compartment	Volume (L) ^1^	Flow (L/min) ^1^	Partition Coefficient ^2^	
Alveolar ventilation	-	8.90	Blood:air	1.08
Lungs and arterial blood	1.44	6.32	Lung:blood	1.24
Rapidly perfused tissues (VRG)	2.09	3.20	VRG:blood	1.24
Fat tissues	15.43	0.34	Fat:blood	3.94
Muscles	17.45	1.15	Muscle:blood	1.34
Liver ^3^	1.48	1.64	Liver:blood	0.88

^1^ Volumes and flows were scaled in the Monte Carlo simulations according to Nihlén and Johanson [42]. ^2^ See Ernstgård et al. [41] for sources. ^3^ Metabolism assumed to be zero. Reproduced with permission from [41]; Published by Elsevier, 2014.

**Table 2:** DFE exposure scenarios used in the PBPK simulations.

Subject/Scenario	Inhaled Concentration	Total Abuse Duration	Inhalation Duration	Inhalation Cycle
X	1,000,000 ppm	6 h	1.5–8 s	3–5 min
Y	1,000,000 ppm	2 h	5–15 s	2–5 min

**Table 3:** Reported limits of detection (LOD) and quantification (LOQ) for DFE in blood.

LOD (mg/L)	LOQ (mg/L)	Reference
0.018	0.099	[45]
Ns ^1^	4	[46]
0.066	Ns	[10]
Ns	27	[47]
<2.6	Ns	[48]
Ns	0.14 ^2^	[44]
Ns	5.4 ^3^	[28]

^1^ Ns—not stated. ^2^ Reporting limit. ^3^ Evidence of intentional inhalation.

**Table 4:** Predicted maximum detection times of DFE in blood for different detection limits and the two exposure scenarios.

Detection Limit (mg/L Blood)	Scenario	Maximum Detection Time from End of Abuse Session (h)	
		Median	5th–95th Percentile
0.018	X	18.7	15.0–21.5
	Y	15.7	12.0–18.3
0.14	X	13.5	10.5–15.8
	Y	10.5	7.8–12.8
5.4	X	5.2	4.3–6.3
	Y	2.0	1.0–3.3
