# Erratum: Huet, R.; et al. 1,1-Difluoroethane Detection Time in Blood after Inhalation Abuse Estimated by Monte Carlo PBPK Modeling. *Pharmaceutics*, 2020, *12*, 997

**DOI:** 10.3390/pharmaceutics13010050

**Published:** 2020-12-31

**Authors:** Raul Huet, Gunnar Johanson

**Affiliations:** 1 Department of Psychiatry, Volunteer Faculty, University of Missouri-Kansas City School of Medicine, 2411 Holmes Street, Kansas City, MO 64110, USA; rahuet@sbcglobal.net; 2 Department of Psychiatry, Volunteer Faculty, University of Kansas School of Medicine, 3901 Rainbow Blvd., Kansas City, KS 66160, USA; 3 Integrative Toxicology, Institute of Environmental Medicine, Karolinska Institutet, Box 210, SE-17177 Stockholm, Sweden

The authors wish to make the following corrections to this paper [1]:

The sentence in the abstract “The ranges reflect variability in body mass index and hence amount of body fat” should be rephrased as “The ranges reflect variability in body mass index (and, hence, amount of body fat) and, more so, variable inhalation patterns”.

The authors would like to apologize for any inconvenience caused to the readers by these changes.
