# Correction: Huet, R.; Johanson, G. 1,1-Difluoroethane Detection Time in Blood after Inhalation Abuse Estimated by Monte Carlo PBPK Modeling. *Pharmaceutics* 2020, *12*, 997

**DOI:** 10.3390/pharmaceutics13071037

**Published:** 2021-07-07

**Authors:** Raul Huet, Gunnar Johanson

**Affiliations:** 1 Department of Psychiatry, Volunteer Faculty, University of Missouri-Kansas City School of Medicine, 2411 Holmes Street, Kansas City, MO 64110, USA; rahuet@sbcglobal.net; 2 Department of Psychiatry, Volunteer Faculty, University of Kansas School of Medicine, 3901 Rainbow Blvd., Kansas City, KS 66160, USA; 3 Toxicology and Risk Assessment, Integrative Toxicology, Institute of Environmental Medicine, Karolinska Institutet, Box 210, SE-17177 Stockholm, Sweden

The authors wish to make the following corrections to this paper [1]:

Text Correction

There was an error in the original article in the Abstract section: “(3) Results: With a detection limit of 0.14 mg/L, the median MDT was estimated to be 10.5 h (5th–95th percentile 7.8–12.8 h) after the 2-h abuse scenario and 13.5 h (10.5–15.8) after the 6-h scenario. The ranges reflect variability in body mass index and hence amount of body fat; (4) Conclusions: Our simulations suggest that the MDT of difluoroethane in blood after abuse ranges from 7.8 to 15.8 h.” 

A correction has been made to the Abstract section: “(3) Results: With a detection limit of 0.14 mg/L, the median MDT was estimated to be 10.5 h (5th–95th percentile 7.8–12.8 h) after the 2-h abuse scenario and 9.5 h (6.5–11.8 h) after the 6-h scenario. The ranges reflect variability in body mass index (and, hence, amount of body fat) and, more so, variable inhalation patterns; (4) Conclusions: Our simulations suggest that the MDT of difluoroethane in blood after abuse ranges from 6.5 to 12.8 h.” 

Text Correction

There was an error in the original article in the Results section in Paragraph 4: “Thus, the median of the MDT ranges widely, from 2.0 h for the shorter exposure scenario (Y) with the highest detection limit, to 18.7 h for the longer exposure scenario (X) with the lowest detection limit.”

A correction has been made to the Results section in Paragraph 4: “Thus, the median of the MDT ranges widely, from 1.2 h for the highest detection limit (scenario X) to 15.7 h for the lowest detection limit (scenario Y).”

Error in Table

In the original article there was a mistake in Table 4 as published:

The corrected Table 4 appears below:

Text Correction

There was an error in the original article in the Discussion section in Paragraph 1: “With a detection limit (LOQ) of 0.14 mg/L, the predicted median MDT is 13.5 h after the 6 h abuse scenario and 10.5 h after the 2 h abuse scenario.” 

A correction has been made to the Discussion section in Paragraph 1: “With a detection limit (LOQ) of 0.14 mg/L, the predicted median MDT is 9.5 h after the 6 h abuse scenario and 10.5 h after the 2 h abuse scenario.” 

Text Correction

There was an error in the original article in the Discussion section in Paragraph 6: “In conclusion, using PBPK modeling and Monte Carlo simulation, we estimate that the MDT of DFE in blood after abuse is on the order of hours, sufficient to allow testing for it even up to 8–16 h after suspected intoxication.” 

A correction has been made to the Discussion section in Paragraph 6: “In conclusion, using PBPK modeling and Monte Carlo simulation, we estimate that the MDT of DFE in blood after abuse is on the order of hours, sufficient to allow testing for it even up to 7–13 h after suspected intoxication.”

The authors would like to apologize for any inconvenience caused to the readers by these changes.

## Figures and Tables

**Table 4:** Predicted maximum detection times of DFE in blood for different detection limits and the two exposure scenarios.

Detection Limit (mg/L Blood)	Scenario	Maximum Detection Time from End of Abuse Session (h)	
		Median	5th–95th Percentile
0.018	X	18.7	15.0–21.5
	Y	15.7	12.0–18.3
0.14	X	13.5	10.5–15.8
	Y	10.5	7.8–12.8
5.4	X	5.2	4.3–6.3
	Y	2.0	1.0–3.3

**Table 4:** Predicted maximum detection times of DFE in blood for different detection limits and the two exposure scenarios.

Detection Limit (mg/L Blood)	Scenario	Maximum Detection Time from End of Abuse Session (h)	
		Median	5th–95th Percentile
0.018	X	14.7	11.0–17.5
	Y	15.7	12.0–18.3
0.14	X	9.5	6.5–11.8
	Y	10.5	7.8–12.8
5.4	X	1.2	0.3–2.3
	Y	2.0	1.0–3.3

## References

[1] Huet R., Johanson G. (2020). 1,1-Difluoroethane Detection Time in Blood after Inhalation Abuse Estimated by Monte Carlo PBPK Modeling. Pharmaceutics.

